# Supplementary material for: Novel Driver Strength Index highlights important cancer genes in TCGA PanCanAtlas patients
Source: PeerJ. 2022 Aug 11;10:e13860. doi: 10.7717/peerj.13860 (PMC9375969; doi:10.7717/peerj.13860)
Supplement: Supplemental Information 2 [file peerj-10-13860-s002.zip › COHORTS/genes plots/detailed/2021_8_16_14_9_distribution_events_detailed_40.pdf]

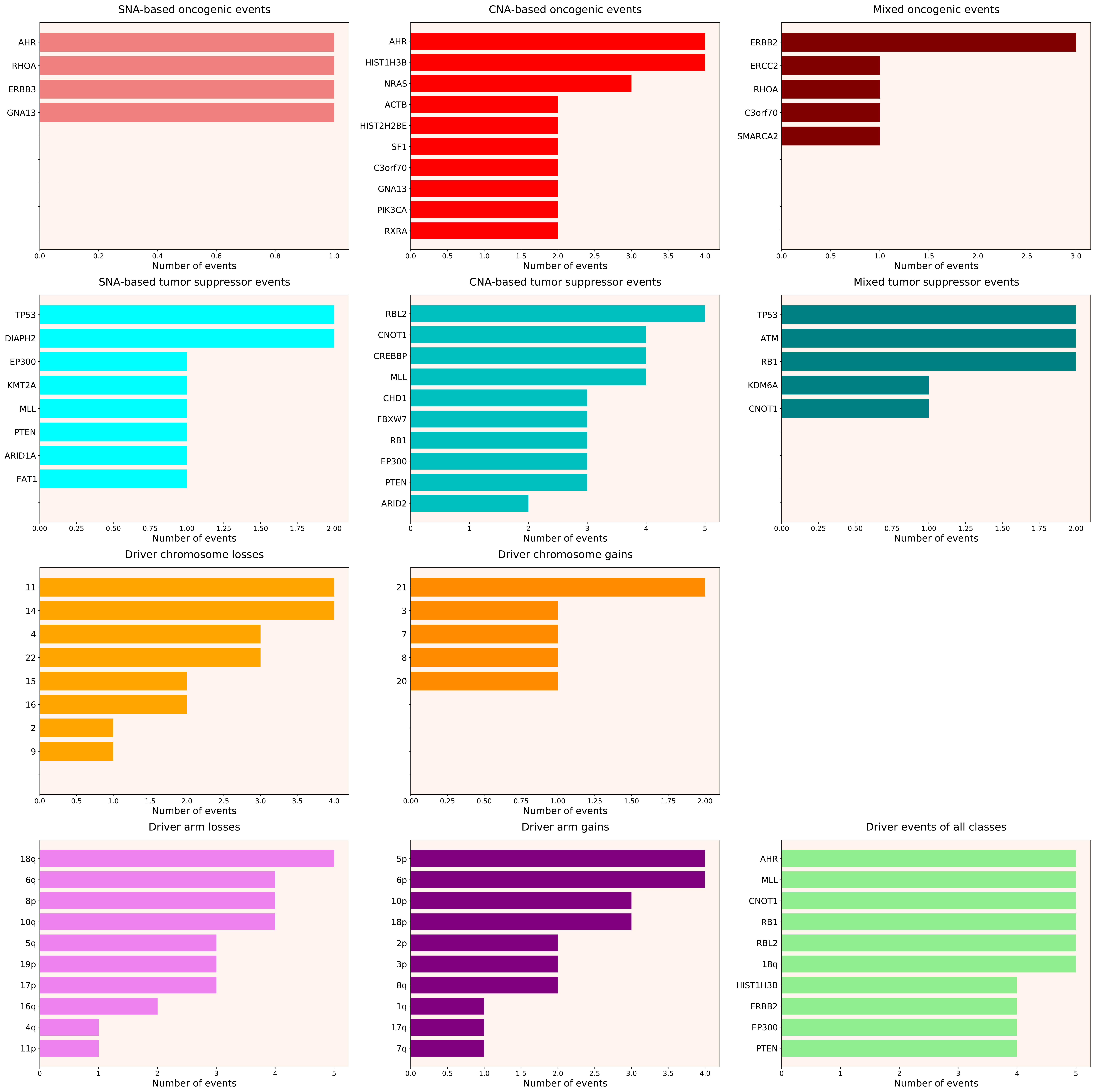

SNA-based oncogenic events
AHR
RHOA
ERBB3
GNA13
0.0 0.2 0.4 0.6 0.8 1.0
Number of events
CNA-based oncogenic events
AHR
HIST1H3B
NRAS
ACTB
HIST2H2BE
SF1
C3orf70
GNA13
PIK3CA
RXRA
0.0 0.5 1.0 1.5 2.0 2.5 3.0 3.5 4.0
Number of events
Mixed oncogenic events
ERBB2
ERCC2
RHOA
C3orf70
SMARCA2
0.0 0.5 1.0 1.5 2.0 2.5 3.0
Number of events
SNA-based tumor suppressor events
TP53
DIAPH2
EP300
KMT2A
MLL
PTEN
ARID1A
FAT1
0.00 0.25 0.50 0.75 1.00 1.25 1.50 1.75 2.00
Number of events
CNA-based tumor suppressor events
RBL2
CNOT1
CREBBP
MLL
CHD1
FBXW7
RB1
EP300
PTEN
ARID2
0 1 2 3 4 5
Number of events
Mixed tumor suppressor events
TP53
ATM
RB1
KDM6A
CNOT1
0.00 0.25 0.50 0.75 1.00 1.25 1.50 1.75 2.00
Number of events
Driver chromosome losses
11
14
4
22
15
16
2
9
0.0 0.5 1.0 1.5 2.0 2.5 3.0 3.5 4.0
Number of events
Driver chromosome gains
21
3
7
8
20
0.00 0.25 0.50 0.75 1.00 1.25 1.50 1.75 2.00
Number of events
Driver arm losses
18q
6q
8p
10q
5q
19p
17p
16q
4q
11p
0 1 2 3 4 5
Number of events
Driver arm gains
5p
6p
10p
18p
2p
3p
8q
1q
17q
7q
0.0 0.5 1.0 1.5 2.0 2.5 3.0 3.5 4.0
Number of events
Driver events of all classes
AHR
MLL
CNOT1
RB1
RBL2
18q
HIST1H3B
ERBB2
EP300
PTEN
0 1 2 3 4 5
Number of events
